# Occurrence of *Spirometra mansoni* in Domestic Dogs from Rural Ecuador and Its Public Health Relevance

**DOI:** 10.3390/ani16132049

**Published:** 2026-07-03

**Authors:** Roberto D. Coello Peralta, Zully Baquerizo Orrala, Aldo Rubén Andrada, Davis Calle Atariguana, Geraldine Ramallo, Alicia Rojas

**Affiliations:** 1 Department of Microbiology, Faculty of Veterinary Medicine and Zootechnics, Universidad de Guayaquil, Guayaquil 090514, Ecuador; davis.callea@ug.edu.ec; 2 Instituto de Genética y Microbiología, Fundación Miguel Lillo, San Miguel de Tucumán T4000JFE, Argentina; arandrada@lillo.org.ar; 3 Consultorio Zu Veterinaria, Comuna San Pablo, Calle 9 de Octubre y Abdón Calderón, Santa Elena 241702, Ecuador; zullybaque-@hotmail.com; 4 Instituto de Invertebrados, Fundación Miguel Lillo, San Miguel de Tucumán T4000JFE, Argentina; gramallo@lillo.org.ar; 5 Laboratory of Helminthology, Centro de Investigación en Enfermedades Tropicales, Universidad de Costa Rica, San José 2060, Costa Rica; anaalicia.rojas@ucr.ac.cr

**Keywords:** *Spirometra mansoni*, coproparasitic techniques, morphometry, PCR, domestic dogs, epidemiology, public health

## Abstract

*Spirometra mansoni* causes sparganosis in humans and spirometrosis in domestic dogs. It is transmitted through the consumption of raw or undercooked meat from fish, frogs or paratenic animals, producing subcutaneous and tissue infections in humans, whereas dogs or cats develop gastrointestinal infections. Sparganosis is a zoonotic parasitosis associated with freshwater aquatic environments, prevalent in tropical and subtropical regions of the world. The purpose of this investigation was to identify *S. mansoni* in domestic dogs from riverine sectors of the Daule River in Ecuador, using coproparasitological methods: direct examination, flotation and sedimentation with centrifugation using saline solution (as screening); and for confirmation, morphometric methods and PCR were used. Through a descriptive, prospective and cross-sectional study, 402 domestic dogs were analyzed, and *Spirometra mansoni* were determined in 67 (16.66%) of the collected samples. The clinical and epidemiological characteristics of spirometrosis in dogs and the potential risk of sparganosis occurring in humans were determined. However, cases may appear in pets from other locations.

## Simple Summary

*Spirometra mansoni* causes sparganosis, a parasitic disease affecting human tissues, and spirometrosis in dogs and cats, which can cause gastrointestinal disorders; both pathologies are of animal and public health concern. Freshwater fish and amphibians play a crucial role in the spread of this parasite in the environment. However, there is no information on infection in dogs in Ecuador. In this study, we examined fecal samples from 402 dogs from riverside areas along the Daule River (Ecuador) to confirm the presence of the parasite. We used several coproparasitological techniques as screening methods, followed by morphometry and PCR for confirmation, indicating exposure to this tapeworm. A total of 16.66% (67/402) of the dogs tested showed evidence of infection, suggesting that the parasite circulates among dogs in these areas. We also analyzed clinical and epidemiological characteristics of spirometrosis in dogs and the risk of sparganosis in humans. Our findings provide new information on the presence of this parasite in dogs living in rural environments in Ecuador and can contribute to improving disease surveillance and control strategies to reduce the risk of infection in canines, felines and humans.

## 1. Introduction

*Spirometra* is a genus of pseudophyllid cestodes that cause an anthropozoonotic disease. The biological life cycle of this parasite begins when adult *S. mansoni* located in the small intestine of their definitive hosts, usually domestic cats and dogs, lay eggs that reach freshwater [1]. The eggs mature and release the coracidia (ciliated mobile larvae), which are then ingested by copepod first intermediate hosts of the genus *Cyclops*, where procercoid larvae develop. Second intermediate hosts, such as fish or amphibians, ingest infected copepods and develop plerocercoid larvae. The latter are the infective forms for definitive hosts, accidental hosts (such as humans) and paratenic hosts (such as reptiles, birds and mammals including rodents, bears, pigs and monkeys) [2]. In the final definitive hosts, plerocercoid larvae reach the digestive tract, and the adult parasite develops in the small intestine, producing spirometrosis [1,2], whereas in accidental hosts (humans) larval development is halted in hosts and sparganosis develops.

Sparganosis is a metacestodiasis or larval cestodiasis caused by *S. mansoni* infection [1]. This tapeworm is transmitted to humans by the ingestion of copepods infected with procercoid larvae in natural water, the consumption of fish and amphibians (like tadpoles and frogs), or the consumption of undercooked meat, viscera or blood from paratenic animals containing plerocercoid larvae [3,4]. These larvae can reach several centimeters and live for up to 20 years in humans. The lesions can give rise to granulomas that can transform into abscesses and produce clinical manifestations depending on the place where they are finally located [2]. Plerocercoid larvae in humans have been described with greater frequency in subcutaneous tissues as palpable, fixed, or migrating painless masses, associated with redness or itching. In addition, plerocercoid larvae can be found in other anatomical sites, such as the abdominal wall, chest wall, lower limbs, scrotum, pleural cavity, lungs, eye, orbit, abdominal viscera, and nervous system [1,2]. The most serious manifestation is cerebral sparganosis, which is accompanied by headache, hemiparesis, hemianopia, and seizures [2].

Currently, five species of *Spirometra* are recognized worldwide: *S. erinaceieuropaei*, *S. decipiens*, *S. mansoni*, *S. ranarum*, and *S. mansonoides* [1,2]. However, the species with the greatest epidemiological importance is *S. mansoni*, which is distributed on several continents, including Europe, Asia, Africa and Australia [2], with the global incidence of infection between 0.089% and 0.313% [3]. In addition, human cases of sparganosis for *S. mansoni, S. erinaceid*, *S. ranarum*, and *S. proliferum* have been reported in China, Japan, Taiwan, Korea, Vietnam, Thailand and other countries in Southeast Asia and the Western Hemisphere after the consumption of intermediate hosts infected with procercoid or plerocercoid larvae [2].

*Spirometra mansoni* infection in domestic dogs and cats is usually asymptomatic but can lead to diarrhea, vomiting, weight loss, and enteritis [5]. *S. mansoni* has been reported in Canada [6], the United States [2], Mexico [6], Puerto Rico, Costa Rica [7], Honduras, Belize, the Cayman Islands [6], Cuba [7], Grenada, the Netherlands Antilles, Venezuela, Colombia [6], Ecuador [8], Brazil [9], Bolivia [10], Paraguay [2], Uruguay and Argentina [10].

In Ecuador, a natural infection of *S. mansoni* was described in 1974 in a domestic cat determined by morphometry of the adult parasite [8], and in 1990, nodulation located in the left scapular region was identified in a human, which, after its extirpation, revealed the presence of a cestode classified morphometrically as *Spirometra* sp. [11].

On the other hand, the internal transcribed spacer 2 (ITS2) is a non-coding locus located between the 5.8S and 28S rRNAs. Phylogenetic studies have shown that it is a highly informative marker in eukaryotes and is widely used for the identification of parasites [12].

**Figure 1:**
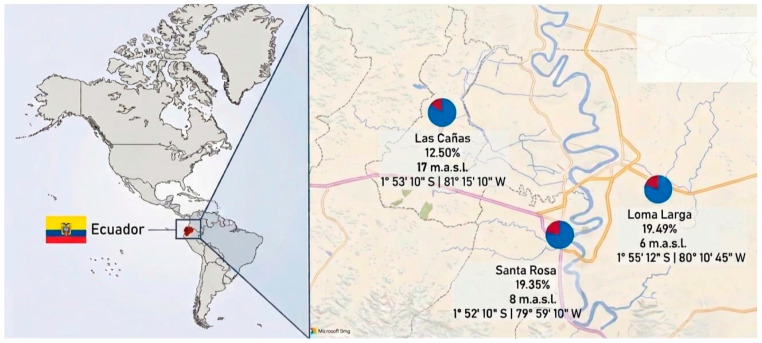
Map with sites of collection and results of *S. mansoni* presence in domestic dogs. Bubble size is proportional to the number of collected samples per region.

Therefore, this study aims to (1) describe this parasite in domestic dogs through fecal analysis, (2) analyze its prevalence in riverine areas of Ecuador (Figure 1), and (3) evaluate the animals’ clinical characteristics, the epidemiology of the parasite, and its implications for public health.

## 2. Materials and Methods

### 2.1. Selection of the Areas and Time of the Study

The sites studied were the riverine areas of the Daule River, including Loma Larga in the city of Nobol; Santa Rosa in the city of Daule; and Las Cañas in the city of Lomas de Sargentillo; all located in the province of Guayas on the Ecuadorian coast (Figure 1). The areas studied were selected because cases of dogs with gastrointestinal pathologies appeared at the Besito Vet Pet veterinary clinic in the city of Guayaquil (Ecuador), coming from the aforementioned sectors. This research was conducted from 1 March to 30 September 2023.

### 2.2. Sample Size Calculation and Inclusion and Exclusion Criteria

An average of 5 domestic dogs per household (3650 dogs) live in this area. With this information, sample size was calculated using the online program Qualtrics ^XM^ (Seattle, WA, USA), with a confidence level of 95%, a sampling error of 5% and a population size of 3650 domestic dogs. A sample size of 348 dogs was estimated, and in this study, a total of 402 domestic dogs were analyzed [13].

The inclusion criteria used in this research were: (1) Owners of domestic dogs residing in the studied areas who provided informed consent to participate in the research and for the study of their dogs. (2) Owners with two or more domestic dogs. (3) Individuals who have frequent contact with the household dog. (4) Sampling of individuals and their domestic dogs of all ages. (5) Properly obtained, labeled, preserved, and analyzed fecal samples. Any individual or dog that did not meet these criteria was excluded from the study.

### 2.3. Recognition of the Sectors Studied, Survey and Physical Verification of Participants

With the help of local residents, we inspected the areas and explained to the population the importance of the research, as well as the potential risk of contracting sparganosis.

A targeted survey was conducted with the individuals who decided to participate in the study prior to informed consent. The questions included issues such as knowledge of sparganosis, awareness of cases, consumption of exotic foods (fish, frogs, tadpoles, copepods, snakes, birds and pigs), and consumption of untreated water. Participants were also asked about their dogs’ food and drinking water consumption, contact with the river, ownership of domestic dogs, deworming of animals, the dogs’ habitat, and veterinary care.

The physical verification of domestic dogs was carried out, and the following parameters were evaluated: body temperature; body weight (low, medium and high) through weighing; body condition (scale: overweight, good, fair and poor) following the guidelines established by the World Association of Small Animal Veterinarians (WSAVA); and muscle mass (scale: 1: severe muscle wasting; 2: mild-to-moderate muscle wasting; 3: normal muscle); conditions of the skin, coat, and ocular mucosa; and signs of disease [14].

Additionally, the dog owners underwent physical examinations to look for granulomas or abscesses; they were also asked about any medical diagnoses or if they knew anyone in their circle who had the condition. Finally, the data were recorded [15].

### 2.4. Collection, Transportation and Analysis of Samples from Domestic Dogs

Fecal samples of domestic dogs were collected by non-probabilistic convenience sampling in riverside sectors of the Daule River (province of Guayas, Ecuador) (Figure 1).

To obtain fecal samples from domestic dogs, sterile jars were used, and the following data were recorded: address, owner name, and telephone contact. In cases where the owner of the animal could not collect a sample from their pet, a technical team carried it out following the procedures of Dubie et al. [16], collected directly from the rectum of the dogs.

Fecal samples from dogs were stored in refrigerated coolers between 4 and 8 °C and transported to the Besito Vet Pet Lab laboratory in the city of Guayaquil (Ecuador) for subsequent analysis using coproparasitological methods [17].

### 2.5. Coproparasitological Methods

All samples were immediately analyzed via the following coproparasitic methods: direct examination, flotation (Willis), and sedimentation with centrifugation using saline solution (to increase the sensitivity and specificity of the screening). The samples were stained with Lugol’s iodine solution, then observed using optical microscopy with a magnification from 40× to 400× [18,19].

Additionally, fecal and blood samples were collected from animals positive for *S. mansoni*. To rule out co-infections, immunochromatographic assays were performed to detect viral antigens (*Canine parvovirus* and *Canine distemper Virus*), bacterial antigens (*Ehrlichia canis* and *Anaplasma* sp.), and parasitic antigens (*Babesia* sp., *Leishmania* sp., and *Dirofilaria immitis*). Additionally, acid-fast bacilli (AFB) and lactophenol cotton blue staining were used for bacterial and fungal screening, respectively.

### 2.6. Diagnostic Criteria and Morphometry

For the identification of *S. mansoni* eggs the following criteria described by Bowman [19] and Alvarado et al. [7] were considered: eggs capped at a distal end of the egg with a flattened shape, equatorial bulge, brown in color, between 60 and 70 µm long and 30–40 µm wide; elliptical, rounded or convex in shape and the presence of morulated structures inside.

### 2.7. DNA Extraction and PCR

The positive samples identified by the screening methods were processed using 0.5 g of sample in 500 μL of DNA/RNA Shield solution at the Besito Vet Pet laboratory. They were then transported at −20 °C to the Institute of Genetics and Microbiology of the Miguel Lillo Foundation (San Miguel de Tucumán, Argentina), where the eggs were lysed following the procedures described by Coello et al. [20]. DNA extraction was subsequently performed via the Qiagen DNeasy blood and tissue kit. The sequences of the ITS2 intron were amplified via the specific primers Sman-F (5′-CGC CTA ATA AAA CAG CCG GC-3′) and R (5′-GTT CAG CGG GTA ATC TCG ACT-3′). A sample provided by Dr. Marcos Javier Butti of the Laboratory of Human Parasitosis and Zoonosis Parasitarias of the National University of La Plata (Argentina) was used as a positive control.

The PCRs were conducted in a final volume of 25 μL, which included: 2 μL of genomic DNA template (100 ng/μL), 0.3 μL of 2U TAQ T-plus High Way polymerase, 2.5 μL of Taq 10 × High Way Buffer, 1.5 μL of 25 mM MgCl_2_ from Thermo Scientific (Invitrogen Corp., Buenos Aires, Argentina), 0.5 μL of primers, 1 μL of 0.5 mM dNTPs, and 16.7 μL of ddH_2_O. Amplification was performed in a Nyxtecnik^®^ (San Diego, CA, USA) thermocycler using the following PCR profile: 1 cycle at 94 °C for 5 min, followed by 36 cycles at 94 °C for 40 s, 46 °C for 40 s, and 72 °C for 1 min and 10 s, with a final step of 1 cycle at 72 °C for 5 min and a hold at 4 °C. The PCR products were analyzed by electrophoresis on 1.5% agarose gels stained with GelRed Nucleic Acid Gel Stain (10,000×) and visualized via a UV transilluminator.

The PCR products were sequenced by Macrogen, Inc. (Seoul, Republic of Korea).

### 2.8. Sequence Identity and Alignment

Ten positive samples were selected per area investigated for the phylogenetic study. Since no differences were found between the sequences obtained, two samples per sector were analyzed and compared with DNA obtained from adult parasites (positive control), as described by Alvarado et al. [7]. A BLAST search [http://blast.ncbi.nlm.nih.gov/Blast.cgi (accessed on 15 April 2026)] was also performed, and the results were verified against those registered in GenBank to confirm the species identity (BLAST+2.17.0). DNA sequences were automatically aligned using SeaView 4 [21], and subsequently manual alignment and comparison were performed using MEGA 6 [22].

### 2.9. Molecular Data Analysis

The online tool FaBox was used to determine the presence of haplotypes in the analyzed ITS2 sequences. For phylogenetic analysis, reference ITS2 sequences from several *Spirometra* species were retrieved from the GenBank database. Only sequences exhibiting 100% coverage of the amplified segments were included in the analysis (Table 1). Sequences of *Diphyllobothrium schistochilos* and *Dibothriocephalus nihonkaiensis* (GenBank accession numbers MW601833.1 and MW465840.1, respectively) were selected as outgroups. Maximum parsimony (MP) analyses were performed using MEGA 6 software. To avoid potential bias caused by overrepresentation, only one sequence per haplotype was included in the molecular phylogenies.

### 2.10. Statistical Analysis

The prevalence of *Spirometra mansoni* was estimated for each sampling site and weight category as the proportion of positive dogs out of the total number of animals examined, expressed as a percentage. Exact 95% confidence intervals (95% CIs) were calculated using the Clopper–Pearson exact binomial method. Differences in prevalence between Santa Rosa, Loma Larga, and Las Cañas were initially assessed using a χ^2^ test of independence applied to a 3 × 2 contingency table (site × parasite status). Fisher’s exact test was also performed for paired comparisons between locations (Santa Rosa–Loma Larga, Santa Rosa–Las Cañas, and Loma Larga–Las Cañas). Odds ratios (ORs) and their respective 95% confidence intervals were estimated for each comparison. In order to control for the increase in Type I error associated with multiple contrasts, the *p*-values obtained in paired comparisons were adjusted using Holm’s sequential procedure.

The association between the presence of *Spirometra mansoni* and the explanatory variables (weight category and place of origin) was assessed using binary logistic regression. A penalized logistic regression model [23,24] (Firth, 1993; Heinze and Schemper, 2002) was also employed. An additive model was fitted that included weight category and place of origin as covariates. Results are expressed as odds ratios (ORs), 95% confidence intervals, and *p*-values.

In all analyses, a significance level of α = 0.05 was considered. Statistical analyses were performed using R software version 4.6.1 [25]. Binomial confidence intervals were estimated using the binom package (version 1.1-1.1) and Firth’s penalized logistic regression was implemented via the logistf package (version 1.26.1) [26].

## 3. Results

### 3.1. Prevalence and Epidemiology of S. mansoni in Domestic Dogs from Riverine Sectors of the Daule River

The overall prevalence of this parasite in domestic dogs was 16.66% (67/402). Frequencies varied significantly among the sampled areas (*p* < 0.05 and Student’s t test), with rates of 19.49% (23/118; CI 95%: 12.78–27.80), 19.35% (24/124; CI 95%: 12.81–27.42), and 12.50% (20/160; CI 95%: 7.81–18.64) detected in dogs from Loma Larga, Santa Rosa, and Las Cañas, respectively (Table 2). The comparison of prevalences between sites using a χ^2^ test of independence showed no significant differences (χ^2^ = 2.823, df = 2, *p* = 0.244). Fisher’s exact tests with Holm’s correction also showed no significant differences in the paired comparisons between the analyzed sites. This is the first report of *Spirometra mansoni* in domestic dogs in Ecuador.

### 3.2. Microscopic Identifications Using Coproparasitological Screening Techniques and Morphometry

**Figure 2:**
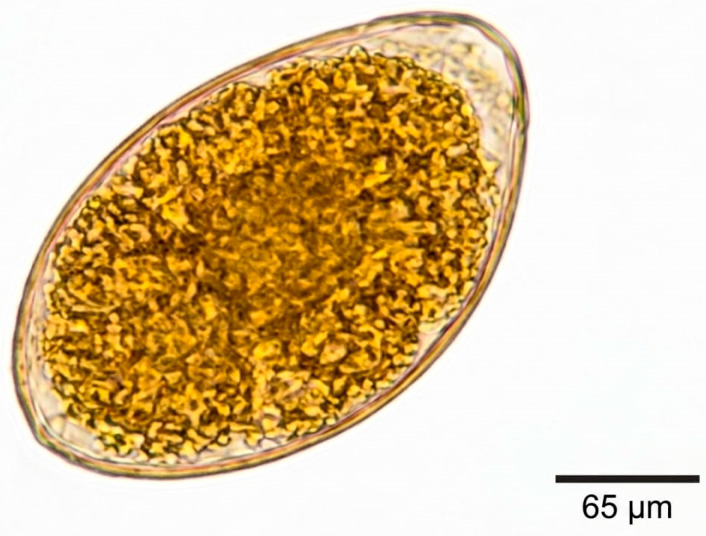
The eggs of *S. mansoni* in the feces of domestic dogs were identified through sedimentation by centrifugation in saline solution, and the eggs were observed by optical microscopy at 40× and dyed and fixed with Lugol.

During the coprological analysis of fecal samples from dogs, eggs with a brown cap were observed, averaging 60 to 70 µm in length and 30 to 40 µm in width. The eggs had equatorial bulges and the operculum was located at a distal end of the egg with a flattened shape. Some eggs were elliptical, whereas others were more rounded or convex. Additionally, morulated structures were observed inside the eggs as described by Alvarado et al. [7] (Figure 2).

### 3.3. Identification and Molecular Phylogeny Using the ITS2 Marker

Although mature proglottids and adult parasites could not be recovered, the species was confirmed by egg morphometry and molecular detection of the parasite’s DNA by PCR from fecal samples, identifying canine infections by *S. mansoni*.

Of the 67 positive samples, six (two per study area) underwent PCR using specific primers. All DNA samples obtained yielded a PCR product ranging from 228 to 231 bp (Genbank accession numbers PZ510950.1, PZ510951.1, PZ510952.1, PZ510953.1, PZ510954.1 and PZ510955.1). BLAST analysis of the sequenced amplicons revealed 100% coverage and identity with the *S. mansoni* ITS2 sequences (GenBank accession numbers OK235496.1 and OK235497.1). Furthermore, this analysis revealed 16 Asian sequences with 100% coverage that matched the amplified segment: four belonging to *S. mansoni* and 12 to *S. erinaceieuropaei*.

Haplotype comparison identified three haplotypes for *S. mansoni* and ten for *S. erinaceieuropaei* (detailed in Table 1).

**Figure 3:**
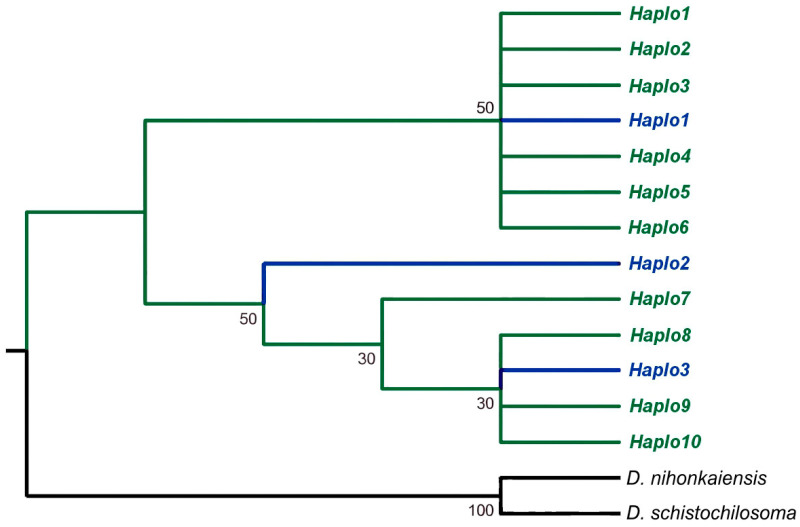
Majority consensus phylogenetic tree obtained via the maximum parsimony analysis for the ITS2 sequence. The blue lines indicate *Spirometra mansoni*, green lines represent *S. erinaceieuropaei*, and gray lines represent the outgroup. The numbers indicate bootstrap values. IC = 1.00, IR = 0.9999.

The MP analysis resolved two main clades (bootstrap = 50%): Clade A, which includes *S-man-Haplo1* and *S-eri-Haplo1* to *S-eri-Haplo6*; and Clade B, which clusters *S-man-Haplo2* and *S-man-Haplo3* with *S-eri-Haplo7* to *S-eri-Haplo10*. The Guayas samples analyzed in this study corresponded to *S-man*-*Haplo3*, nesting closely with the Asian accessions *S-eri-Haplo8* (FJ886751.1) and *S-eri-Haplo10* (FJ886749) from China, and *S-eri-Haplo9* (OQ449433.1) from Cambodia (Figure 3).

### 3.4. Determination of Clinical Characteristics in Domestic Dogs from the Studied Sectors

All domestic dogs studied were of mixed breed, aged between 1 and 10 years, with males (60%) and females (40%).

Physical examination of the 402 domestic dogs revealed that 385 (96%) had normal body temperatures, whereas 17 (4%) had high body temperatures. In addition, low weight showed a prevalence of 20.2% (37/183; 95% CI: 14.7–26.8), the medium weight category showed a prevalence of 24.6% (30/122; 95% CI: 17.2–33.2), while in the high weight category no positive dogs were recorded (0/97; 95% CI: 0–3.71); 60% of the dogs had poor body conditions, 30% had fair body conditions and 10% had good body conditions; no significant differences were found. Regarding muscle mass, 60% of the dogs had severe muscle wasting, 30% had mild-to-moderate muscle wasting, and 10% had normal muscle mass. Ninety percent of the animals presented dull skin and coat conditions with pale ocular mucous membranes. Finally, 17 animals (4%) presented vomiting, diarrhea, decay, weakness, severe weight loss, poor body condition and pale mucous membranes, which may be consequence of *S. mansoni* infection or other vector-borne pathogens distributed in the region such as *Ehrlichia canis*, *Anaplasma* spp., *Leishmania* spp. or *Dirofilaria immitis*.

When analyzing prevalence by weight category, a more pronounced pattern with significant differences was observed (χ^2^ = 26.577, df = 2, *p* = 1.694 × 10^−6^). The low weight category had a prevalence of 20.2% (37/183; 95% CI: 14.7–26.8), the medium weight category had a prevalence of 24.6% (30/122; 95% CI: 17.2–33.2), while no positive dogs were recorded in the high weight category (0/97; 95% CI: 0–3.71). Paired comparisons showed no significant differences between low and medium weights (OR = 0.78; 95% CI: 0.43–1.40; adjusted Holm *p* = 0.398). In contrast, comparisons between low weight vs. high weight and medium weight vs. high weight were significant, due to the total absence of positives in the high weight category.

Firth’s penalized logistic regression confirmed this pattern (likelihood ratio test = 40.106, *p* = 4.116157 × 10^−8^, n = 402, Wald test = 97.388, *p* = 0). In the additive model that included weight and site, the middle weight category did not differ significantly from the low weight category (OR = 1.34; 95% CI: 0.77–2.32; *p* = 0.298). In contrast, the high weight category showed markedly lower odds of infection compared to the low weight category (OR = 0.019; 95% CI: 0.0002–0.141; *p* < 0.001). This result indicates a strong association between the high weight category and the absence of *S. mansoni* infection. On the other hand, the localities of Loma Larga (OR = 0.64; 95% CI: 0.33–1.23; *p* = 0.180) and Las Cañas (OR = 0.67; 95% CI: 0.34–1.32; *p* = 0.251) did not differ significantly from Santa Rosa after adjusting for weight category.

### 3.5. Determination of Clinical Characteristics in People from the Studied Sectors

People interviewed (177 persons) were unaware of the infection caused by *Spirometra* sp. and of any sparganosis infections that may have occurred in the last five years. Forty percent said they consumed some type of exotic animal (fish, frogs, tadpoles, copepods, snakes, birds, or pigs) with little or no cooking, 60% consumed untreated water, and each family owned between six and seven domestic dogs. Moreover, the owners of the dogs stated that all animals consumed untreated food and water. In addition, all people and dogs had contact with the river, and none of the dogs were dewormed or received veterinary care, living in intra- and peridomestic habitats. Moreover, all studied sites were rural, where there were no sewerage facilities or treatment plants to purify drinking water.

No cases of subcutaneous lesions suggestive of sparganosis were recorded during the physical examination of subjects.

## 4. Discussion

The prevalence of 16.66% obtained is higher than that reported globally (0.089% and 0.313%) and that from Asia (0.696%), Africa (0.224%), and Oceania (0.203%) [3]. However, prevalences ranging from 0% to 77.9% have been reported in domestic dogs in China [4]. These findings suggest that the animals sampled in this study are highly exposed to paratenic and second intermediate hosts carrying larval stages of *S. mansoni* and thus become infected with the parasite, either by feeding or hunting habits.

The eggs identified by the different coproparasitological and morphometric methods had characteristics that coincided with those reported for *S. mansoni* [7,19].

In the Americas, *S. mansoni* has been reported in: frogs and domestic cats in the United States [6]; domestic cats in Mexico [27]; coyotes, dogs, and domestic cats in Costa Rica [7]; foxes (*Cerdocyon thous*) in Colombia [28]; and domestic cats in Ecuador [8]. Additionally, *S. decipiens* has been reported in a human in Venezuela, in snakes and field foxes in Brazil [6], in a fox in Bolivia [6], in a domestic cat in Chile, and in wild cats in Argentina [28]; and *S. proliferum* has been reported in humans in Venezuela [29].

Ecuador is a tropical country located in western South America, and has four regions with a variety of climates (Coastal, Andean, Amazonian, and Insular) [30]. Neglected freshwater zoonotic parasitic diseases, such as opisthorchiasis, diphyllobothriasis, alariasis, gnathostomiasis, and sparganosis, can occur in tropical coastal areas [31].

This is the first report of the presence of *S. mansoni* in domestic dogs from riverside sectors of the Daule river on the Ecuadorian coast, and its clinical, epidemiological, and public health implications. Moreover, in Ecuador, *S. mansoni* has been previously described in domestic cats [8] and in humans [11], suggesting that the parasite has been present in the country for an indefinite period of time. Furthermore, it is important to highlight that the sites studied had temperatures ranging from 20 °C to 37 °C, with a tropical savanna climate.

On the other hand, although there are few studies on the molecular identification of *S. mansoni* in domestic dogs using the ITS2 molecular marker, Zendejas-Heredia et al. [32] described its use in 15 dogs from Cambodia. This approach differs from other studies that use the COX1 molecular marker, as described by Alvarado et al. [7] in Costa Rica, Salazar et al. [24] in Mexico, and Yamasaki et al. [33] in Japan.

Despite haplotype variability, the resulting phylogeny resolved two main clades that mostly exhibited a comb-like structure or unresolved polytomies, likely due to low genetic diversity among the analyzed geographic origins. Interestingly, the GenBank accession KC561781.1 (specifically annotated as *S. erinaceieuropaei*) showed a 100% sequence identity with *S-man-Haplo3* within the analyzed fragment, suggesting a potential taxonomic misidentification in the database. Conversely, accession OK235499.1 (*S-man-Haplo1*) harbored specific nucleotide insertions (CCG at positions 103–105 and GTGGCG at positions 160–165) typical of *S-eri-Haplo5*. This signature placed it firmly within Clade A, positioned far from the remaining *S. mansoni* sequences.

Likewise, the domestic dogs studied were of mixed breeds, with ages between 1 and 10 years. However, Yamasaki et al. [33] and Muñoz [2] described that any domestic dog of any age can be susceptible to spirometrosis.

With regard to the clinical characteristics determined in the present study, they differ from those reported by Coello et al. [30], but are similar to those explained by Bowman [19] and Alvarado et al. [7].

In addition, it is important to note that there is little reference on this subject, as infection rates in dogs vary from 1% to 33% [34]. Other reports have shown that infected animals can be asymptomatic, whereas others can present with enteritis, diarrhea, vomiting and weight loss [5,19,35]. In this sense, further studies analyzing blood samples from dogs should be conducted to determine the true morbidity associated with *S. mansoni* infections, as well as to decipher the prevalence of these other pathogens in dogs from endemic regions.

The identified socio-epidemiological factors influenced the occurrence of 16.66% of parasitic infection in domestic dogs; however, cases of sparganosis in humans could also occur. This is similar to the findings described by Coello et al. [30], Badri et al. [3], and Kavana et al. [36]. However, these findings differ from those of Hong et al. [4], who described a prevalence of socio-epidemiological variables that increases the risk of parasitic infection to 27.5%. These results demonstrate that the human population in the three studied sectors could become infected with this parasite at any time due to cultural and dietary habits, as well as a general lack of knowledge about the infection.

The results of this study indicate that the prevalence of *S. mansoni* did not vary significantly between locations, but it did show a marked association with weight category. The absence of positive dogs in the high weight category suggests that the infection was concentrated in low- and medium-weight animals. However, given the cross-sectional nature of the study, this result should be interpreted as an association and not as evidence of causality; that is, it does not allow us to establish whether the infection contributes to a lower body condition score or whether lower-weight dogs are more susceptible to infection. These results differ from those described by Coello et al. [30] and Tan et al. [37].

No cases of sparganosis were reported in humans. However, Muñoz [2], Lin et al. [34], Muigg et al. [38] and Yim et al. [39] described sparganosis in the abdominal wall, chest wall, lower limbs, scrotum, pleural cavity, lungs, eye, orbit, abdominal viscera and nervous system of human patients. Therefore, sparganosis-associated lesions may not have been properly identified. For this purpose, it would be ideal to carry out serological tests that evaluate the antigens of the parasite in individuals, providing insights into their contact with dogs infected with the parasite. Similarly, bacterial, fungal or viral infections were ruled out in domestic dogs, resulting in negative results in cultures, stains and serological tests for parvovirus and canine distemper.

Among the study’s limitations, it was noted that, due to a limited budget, the sampling strategy differed. Furthermore, PCR testing could not be performed on all samples, and a more in-depth diagnostic interpretation of the human health-related observations was lacking.

## 5. Conclusions

Spirometrosis in pets and sparganosis in humans are underdiagnosed freshwater parasitic infections in Ecuador. This research is the first report of *S. mansoni* in domestic dogs in Ecuador, with a prevalence of 16.66%. Coproparasitological methods were used to identify parasite eggs: direct examination, flotation, and sedimentation with centrifugation in saline solution (as a screening method) using an optical microscope, followed by confirmation through morphometry and PCR. Clinical and epidemiological characteristics influencing the moderate prevalence of this parasite in domestic dogs were identified; however, no cases of sparganosis in humans were found.

Although the ITS2 marker is widely employed for parasite identification, it remains underrepresented in genetic databases for the genus *Spirometra*. Consequently, expanding the number of sequences for *S. mansoni* is highly valuable for elucidating intraspecific genetic diversity and ensuring accurate species identification.

Statistically, a significant difference was observed between the prevalence of *S. mansoni* and the body weight of domestic dogs.

The results of this research highlight the potential risk of zoonotic transmission to humans due to the presence of *S. mansoni* in the region’s definitive hosts, frequent human–animal contact, and a lack of awareness among the local population regarding this parasitic infection. Therefore, it is important to educate the population about this parasite from a One Health perspective, implement sanitary measures, deworm animals regularly, and prevent the consumption of raw or undercooked meat, fish, and alternative hosts.

## Figures and Tables

**Table 1:** Shared haplotypes: list of species and GenBank accession numbers of the sequences of the same haplotype. The sequences used in the maximum parsimony analysis are indicated in bold. Asterisks (*) indicate sequences generated in this study.

Specie	Haplotype	GenBank Accession	Host	Country
*Spirometra mansoni*	*S-man-Haplo1*	**OK235499.1**	Cat	China
	*S-man-Haplo2*	**OK235498.1**	Cat	China
	*S-man-Haplo3*	OK235497.1	Cat	China
		OK235496.1	Cat	China
		KC561781.1	Cat	China
		**PZ510950**.1 *	Dog	Ecuador
		PZ510951.1 *	Dog	Ecuador
		PZ510952.1 *	Dog	Ecuador
		PZ510953.1 *	Dog	Ecuador
		PZ510954.1 *	Dog	Ecuador
		PZ510955.1 *	Dog	Ecuador
*S. erinaceieuropaei*	*S-eri-Haplo1*	**FJ886746.1**	Dog	China
	*S-eri-Haplo2*	**FJ886753.1**	Dog	China
	*S-eri-Haplo3*	**FJ886748.1**	Dog	China
	*S-eri-Haplo4*	**OQ449434.1**	Dog	Cambodia
		OQ449435.1	Dog	Cambodia
		FJ886754.1	Dog	China
		FJ886755.1	Dog	China
	*S-eri-Haplo5*	**FJ886747.1**	Dog	China
	*S-eri-Haplo6*	**FJ886752.1**	Dog	China
	*S-eri-Haplo7*	**FJ886750.1**	Dog	China
	*S-eri-Haplo8*	**FJ886751.1**	Dog	China
	*S-eri-Haplo9*	**OQ449433.1**	Dog	Cambodia
	*S-eri-Haplo10*	**FJ886749.1**	Dog	China

**Table 2:** Prevalence of *S. mansoni* in domestic dogs from riverine sectors of the Daule River, Ecuador.

Riparian Sectors of the Daule River	No. of Animals Studied	Positive Samples	Prevalence	CI 95%
Loma Larga	118	23	19.49%	12.78–27.80
Santa Rosa	124	24	19.35%	12.81–27.42
Las Cañas	160	20	12.50%	7.81–18.64
Total	402	67	16.66%	-

## Data Availability

The data presented in this study are available on request from the corresponding author.

## References

[1] Hyeong-Kyu J., Sun H., Woon-Mok S., Jong-Yil C., Keeseon S.E. (2018). Molecular Genetic Findings of *Spirometra decipiens* and *S. ranarum* in Korea. Korean J. Parasitol..

[2] Muñoz P. (2015). The sparganosis. Rev. Chil. Infectol..

[3] Badri M., Olfatifar M., KarimiPourSaryazdi A., Zaki L., Madeira L., Fasihi M., Barikbin F., Madani P., Vafae A. (2022). The global prevalence of *Spirometra* parasites in snakes, frogs, dogs, and cats: A systematic review and meta-analysis. Vet. Med. Sci..

[4] Hong Q., Feng J., Liu H., Li X., Gong L., Yang Z., Yang W., Liang X., Zheng R., Cui Z. (2016). Prevalence of *Spirometra mansoni* in dogs, cats, and frogs and its medical relevance in Guangzhou, China. Int. J. Infect. Dis..

[5] Adolph C., Peregrini A., Sykes J.E. (2024). *Spirometra masonoides*. Greene’s Infectious Diseases of the Dog and Cat.

[6] Kuchta R., Phillips A.J., Scholz T. (2024). Diversity and biology of *Spirometra* tapeworms (*Cestoda*: *Diphyllobothriidea*), zoonotic parasites of wildlife: A review. Int. J. Parasitol. Parasites Wildl..

[7] Alvarado I., Campos J., Arguedas Y., Romero L.M., Alfaro A., Anchia G., Bass L.G., Berrocal I., Hagnauer I., Olivares R.W. (2024). Molecular, morphological and histopathological evidence of *Spirometra mansoni* in wild and domestic animals from Costa Rica. Vet. Parasitol. Reg. Stud. Rep..

[8] Mueller J.F., Fróes O.M., Fernández T. (1975). On the occurrence of *Spirometra mansonoides* in South America. J. Parasitol..

[9] Silva W.I., Lima E.F., Silva J.O., Alves M.M., Alves C.L.P., Silva A.L.P., Lima J.A., Feitosa T.F., Vilela V.L.R. (2023). Endoparasites in domestic cats (*Felis catus*) in the semi-arid region of Northeast Brazil. Rev. Bras. Parasitol. Vet..

[10] Vettorazzi R., Norbis W., Martorelli S.R., Garcia G., Rios N. (2023). First report of *Spirometra* (*Eucestoda*; *Diphyllobothriidae*) naturally occurring in a fish host. Folia Parasitol..

[11] Guderian R., Roldan J., Guevara A., Chico M. (1990). Human sparganosis in Ecuador: Report of a case in the province of Esmeraldas. Rev. Soc. Bras. Med. Trop..

[12] Martinez-Hernandez F., Sanchez-Aguillon F., Martinez-Ocaña J., Gonzalez-Arenas N.R., Romero-Valdovinos M., Lopez-Escamilla E., Maravilla P., Villalobos G. (2023). Genetic Variability of the Internal Transcribed Spacer and Pyruvate: Ferredoxin Oxidoreductase Partial Gene of *Trichomonas vaginalis* from Female Patients. Microorganisms.

[13] QualtricsXM How to Calculate Sample Size: Ensure Sampling is Correct. https://www.qualtrics.com/es/gestion-de-la-experiencia/investigacion/calcular-tomano-muestra/.

[14] Milstein M.S., Shaffer C.A., Suse P., Marawanaru A., Heinrich D.A., Larsen P.A., Wolf T.M. (2022). A mixed-methods approach to understanding domestic dog health and disease transmission risk in an indigenous reserve in Guyana, South America. PLoS Neglected Trop. Dis..

[15] Liu W., Gong T., Chen S., Liu Q., Zhou H., He J., Wu Y., Li F., Liu Y. (2022). Epidemiology, diagnosis, and prevention of sparganosis in Asia. Animals.

[16] Dubie T., Sire S., Fentahun G., Bizuayehu F. (2023). Prevalence of gastrointestinal helminths of dogs and associated factors in Hawassa city of Sidama region, Ethiopia. J. Parasitol. Res..

[17] Giraldo M.I., García N.L., Castaño J.C. (2005). Prevalence of intestinal helminths in dogs from Quindío province. BioMedica.

[18] Girard R. (2014). Parasitology Manual, Methods for Primary Health Care Laboratories.

[19] Bowman G.D. (2024). Parasitology for Veterinarians.

[20] Coello Peralta R., Andrada A.R., Vinueza R.L., Pazmiño B., Valencia E.D., Rodríguez E., Salazar M., Ramallo G. (2025). Identification of *Ancylostoma caninum* in Domestic Dogs from Ecuador via Various Techniques. Med. Sci. Monit..

[21] Gouy M., Guindon S., Gascuel O. (2010). SeaView version 4: A multiplatform graphical user interface for sequence alignment and phylogenetic tree building. Mol. Biol. Evol..

[22] Tamura K., Stecher G., Peterson D., Filipski A., Kumar S. (2013). MEGA6: Molecular evolutionary genetics analysis version 6.0. Mol. Biol. Evol..

[23] Firth D. (1993). Bias reduction of maximum likelihood estimates. Biometrika.

[24] Heinze G., Schemper M. (2002). A solution to the problem of separation in logistic regression. Stat. Med..

[25] R Core Team (2026). R: A Language and Environment for Statistical Computing.

[26] Heinze G., Ploner M., Dunkler D., Southworth H., Jiricka L., Steiner G. (2025). *logistf: Firth’s Bias-Reduced Logistic Regression*, R Package Version 1.26.1. https://cran.r-project.org/web/packages/logistf.

[27] Šikić A., Gagović E., Rojas A., Sindičić M., Žilić D.J., Naletlić Š., Balić D., Hodžić A., Beck R. (2025). First molecular identification of *Spirometra mansoni* in the golden jackal (Canis aureus) in Croatia. Front. Vet. Sci..

[28] Brabec J., Uribe M., Chaparro J.J., Hermosilla C. (2022). Presence of *Spirometra mansoni,* causative agent of sparganosis, in South America. Emerg. Infect. Dis..

[29] Fredes F., Mercado R., Salas I.P., Sugiyama H., Kobayashi H., Yamasaki H. (2022). Morphological observation and molecular phylogeny of *Spirometra* decipiens complex 1 *(Cestoda*: *Diphyllobothriidae*) found in cat from Chile. Parasitol. Int..

[30] Coello R., Pazmiño B., Salazar M., Parra S., Vinueza L., Pazmiño J., Neira E., Gómez E., Ramallo G. (2025). Ecoepidemiology of *Ancylostoma* spp. in urban-marginal and rural sectors of the ecuadorian coast and prevalence of cutaneous larvae migrans. Med. Sci. Monit..

[31] Menconi V., Lazzaro V.E., Bertola M., Guardone L., Mazzucato V., Prearo M., Bilska-Zajac E., Cortinovis L., Manfrin A., Arcangeli G. (2023). The occurrence of freshwater fish-borne zoonotic helminths in Italy and neighbouring countries: A systematic review. Animals.

[32] Zendejas-Heredia P., Colella V., Huggins L., Schaper R., Schunack B., Traub R. (2023). An Integrated Coproscopic and Molecular Method Provides Insights into the Epidemiology of Zoonotic Intestinal Helminths of Dogs across Cambodia. Transbound. Emerg. Dis..

[33] Yamasaki H., Sugiyama H., Morishima Y., Sako Y. (2024). Molecular identification of *Spirometra* infections in companion animals and wildlife in Japan. J. Vet. Med. Sci..

[34] Rojas A., Salazar E., Beck R. (2026). *Spirometra mansoni*. Trends Parasitol..

[35] Lin Q., Ouyang J.S., Li J.M., Yang L., Li Y.P., Chen C.S. (2015). Eosinophilic pleural effusion due to *Spirometra mansoni* spargana: A case report and review of the literature. Int. J. Infect. Dis..

[36] Kavana N., Sonaimuthu P., Kasanga C., Kassuku A., Al-Mekhlafi H.M., Fong M.Y., Khan M.B., Mahmud R., Lau Y.L. (2016). Seroprevalence of Sparganosis in Rural Communities of Northern Tanzania. Am. J. Trop. Med. Hyg..

[37] Tan X., Zeng Y., Lu S., Abuzeid A.M., Meng Q., Zou Z., Fan K., Liu W. (2026). The Development and Evaluation of a Loop-Mediated Isothermal Amplification (LAMP) Method for the Detection of *Spirometra mansoni* in Dogs. Vet. Sci..

[38] Muigg V., Ruf M.T., Schwarzkopf S., Huang S., Denisjuk N., Stürmann A., Ritzler M., Wampfler R., Poppert S., Neumayr A. (2019). Case report: Human subcutaneous sparganosis in a Thai migrant. Am. J. Trop. Med. Hyg..

[39] Yim J., Kim Y.A., Park J.H., Park H.E., Song H.B., Kim J.E. (2026). Revisiting human sparganosis: A pathologic review from a single institution. J. Pathol. Transl. Med..

